# Thermal magnetic resonance: physics considerations and electromagnetic field simulations up to 23.5 Tesla (1GHz)

**DOI:** 10.1186/s13014-015-0510-9

**Published:** 2015-09-22

**Authors:** Lukas Winter, Celal Oezerdem, Werner Hoffmann, Tessa van de Lindt, Joao Periquito, Yiyi Ji, Pirus Ghadjar, Volker Budach, Peter Wust, Thoralf Niendorf

**Affiliations:** Berlin Ultrahigh Field Facility (B.U.F.F.), Max-Delbrück Center for Molecular Medicine, Berlin, Germany; Physikalisch Technische Bundesanstalt (PTB), Berlin, Germany; Department of Radiation Oncology, Charité Universitätsmedizin Berlin, Berlin, Germany; Experimental and Clinical Research Center (ECRC), a joint cooperation between the Charité Medical Faculty and the Max-Delbrück Center for Molecular Medicine, Berlin, Germany; MRI.TOOLS GmbH, Berlin, Germany

**Keywords:** Thermal magnetic resonance, Magnetic resonance imaging, Hyperthermia, Radio frequency, RF coil technology, Gliablastoma multiforme, Radiation oncology, Thermometry, Targeted drug delivery, Thermal therapies

## Abstract

**Background:**

Glioblastoma multiforme is the most common and most aggressive malign brain tumor. The 5-year survival rate after tumor resection and adjuvant chemoradiation is only 10 %, with almost all recurrences occurring in the initially treated site. Attempts to improve local control using a higher radiation dose were not successful so that alternative additive treatments are urgently needed. Given the strong rationale for hyperthermia as part of a multimodal treatment for patients with glioblastoma, non-invasive radio frequency (RF) hyperthermia might significantly improve treatment results.

**Methods:**

A non-invasive applicator was constructed utilizing the magnetic resonance (MR) spin excitation frequency for controlled RF hyperthermia and MR imaging in an integrated system, which we refer to as thermal MR. Applicator designs at RF frequencies 300 MHz, 500 MHz and 1GHz were investigated and examined for absolute applicable thermal dose and temperature hotspot size. Electromagnetic field (EMF) and temperature simulations were performed in human voxel models. RF heating experiments were conducted at 300 MHz and 500 MHz to characterize the applicator performance and validate the simulations.

**Results:**

The feasibility of thermal MR was demonstrated at 7.0 T. The temperature could be increased by ~11 °C in 3 min in the center of a head sized phantom. Modification of the RF phases allowed steering of a temperature hotspot to a deliberately selected location. RF heating was monitored using the integrated system for MR thermometry and high spatial resolution MRI. EMF and thermal simulations demonstrated that local RF hyperthermia using the integrated system is feasible to reach a maximum temperature in the center of the human brain of 46.8 °C after 3 min of RF heating while surface temperatures stayed below 41 °C. Using higher RF frequencies reduces the size of the temperature hotspot significantly.

**Conclusion:**

The opportunities and capabilities of thermal magnetic resonance for RF hyperthermia interventions of intracranial lesions are intriguing. Employing such systems as an alternative additive treatment for glioblastoma multiforme might be able to improve local control by “fighting fire with fire”. Interventions are not limited to the human brain and might include temperature driven targeted drug and MR contrast agent delivery and help to understand temperature dependent bio- and physiological processes *in-vivo*.

## Background

Glioblastoma multiforme is the most common and most aggressive malign brain tumor. Despite demarcation in computed tomography (CT) or magnetic resonance imaging (MRI) glioblastoma multiforme presents no clear microscopic barriers towards the unaffected brain [1]. This pathology makes a complete surgical resection challenging if not elusive. Recurrences are typically located in the former tumor region and therefore intensification of local treatment is required. Yet, the first therapeutic attempt is to resect the tumor as completely as possible. Radiation therapy adjuvant to resection improves overall survival versus resection alone [2]. Since 2004 the standard of care consists of combined adjuvant chemoradiation using temozolomide [3]. Arguably, the 5-year survival rate after tumor resection and adjuvant chemoradiation is only 10 %, with almost all recurrences occurring in the initially treated site [3, 4]. Attempts to improve local control using a higher radiation dose were not successful [5] so that alternative additive treatments are urgently needed. Interstitial hyperthermia in addition to external beam radiation therapy and brachytherapy of the brain has been demonstrated to improve survival in a randomized trial [6]. This type of treatment is not applicable to all tumor locations within the brain, and did not become accepted in clinical practice due to its invasive nature.

Another thermal therapy approach is nanotherapy, where a dispersion of iron-oxide (magnetite) nanoparticles is injected into the tumor which is subsequently heated in an alternating magnetic field [7]. This therapy was evaluated in combination with a reduced dose of external beam radiation (median dose 30 Gy) in a single-arm study with patients predominantly with recurrent glioblastoma [8]. The treatment was well received and appeared effective. Notwithstanding this clinical success nanotherapy assumes a sufficient coverage of the tumor region by the nanoparticles, which can be difficult under clinical conditions. In addition, the efficiency of the currently available nanoparticles and the frequency of the magnetic field constitute constraints which require further technological improvements. In this light, nanotherapy for glioblastoma is presently considered not to be ready for routine clinical use. Given the strong rationale for the successful use of hyperthermia as part of a multimodal treatment for patients with glioblastoma [9], non-invasive radiofrequency (RF) hyperthermia might significantly improve treatment results, in addition to standard chemoradiation. To evaluate treatment efficiency non-invasive three dimensional thermal dose evaluation is necessary [10]. MRI is of proven diagnostic value with an ever growing number of applications that support thermal therapies [11, 12]. In current clinical regional RF hyperthermia practice MR thermometry (MRTh) is used for spatiotemporal monitoring of temperature and treatment efficiency [13–15]. While the RF transmission used for MRI is commonly performed at a frequency of 64 MHz (*B*_*0*_ = 1.5 T), RF transmission induced heating interventions in this hybrid systems are achieved with RF antennas usually driven at ~100 MHz [16]. The RF wavelength at 100 MHz is approximately 33 cm in brain tissue, which is not suitable to focus the electromagnetic (EM) energy selectively enough to brain tumors with a tumor size of few centimeters. Higher RF frequencies (f > 100 MHz) are suitable to be applied in the head and neck region to perform localized RF hyperthermia [17–20]. Unfortunately, established systems still lack the ability of non-invasive 3D temperature measurement to monitor and control the thermal dose applied in the treated region and in healthy tissue. Realizing this challenge it was demonstrated, that ultrahigh magnetic fields (UHF, *B*_*0*_ ≥ 7.0 T) render an integrated applicator feasible; including a configuration suitable for MRI, MRTh and controlled targeted RF heating utilizing a single transmission frequency of 300 MHz [20]. The applicator employs the proton MR frequency for targeted RF heating and can be used together with commercially available MR systems and multi-channel RF transmission configurations for imaging diagnostics and for RF hyperthermia applications [20]. Such integrated system will be referred to as thermal MR. The early results indicated that this approach is conceptually appealing for a therapeutic application to intracranial lesions since pre-treatment diagnosis and planning, peri-treatment thermal dose control and adaptation and post-treatment evaluation of the treatment efficiency can be performed with a single device. Recognizing this opportunity this work presents physics considerations together with preclinical results derived from a thermal MR applicator driven at 300 MHz. For comparison, applicator designs are proposed which are capable of utilizing even higher RF frequencies (up to 1 GHz). This approach holds the promise to benefit an effective reduction of the achievable thermal hotspot size. To meet this goal electromagnetic field (EMF) simulations are performed in a human voxel model deduced from a healthy volunteer. Physics considerations and RF antenna designs are presented for 300 MHz, 500 MHz and 1 GHz which correspond to 7.0 T, 11.7 T or 23.5 T with an effective wavelength of approximately 13.5 cm, 8.6 cm and 4.5 cm in brain tissue. These efforts are complemented by RF heating experiments conducted at 500 MHz. The preliminary results suggest that such high frequency systems could be used in two configurations. (i) an integrated thermal MR application [20] in conjunction with a 7.0 T, 11.7 T or 23.5 T UHF MR system or (ii) a hybrid configuration [21, 22] using an external RF power amplifier for RF heating together with a conventional MR systems at 1.5 T or 3.0 T. The merits and limitations of physics, technology and clinical applicability of thermal MR are discussed in the context of adjuvant RF hyperthermia treatment of intracranial lesions.

## Methods

### Ethics statement

All imaging studies were performed after due approval by the local ethical committee (registration number DE/CA73/5550/09, Landesamt für Arbeitsschutz, Gesundheitsschutz und technische Sicherheit, Berlin, Germany). Informed written consent was obtained from each volunteer prior to the study. For the *in-vivo* proof-of-concept study at 7.0 T, 1 healthy subject without any known history of neuro- or cardiovascular diseases was included.

### Thermal MR applicator and RF antenna design

**Fig. 1:**
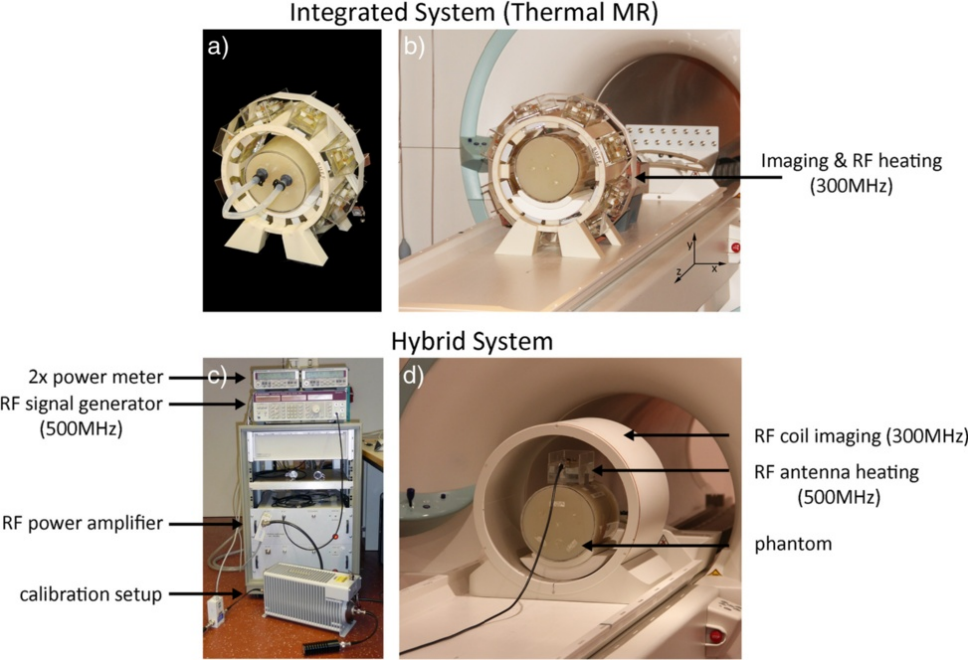
**a**-**b** Experimental setup of an 8-channel thermal MR applicator comprising an array of bow tie dipole antennas that support MR imaging, MRTh and targeted RF heating. **c**-**d** Experimental setup of a hybrid system using an external RF signal generator and RF amplifier at 500 MHz for RF heating, while MRI and MRTh is being performed at 7.0 T with a RF coil operated at 300 MHz. The power amplifier was calibrated to provide an output of 70 W at the end of the feeding cable. A power meter was used to monitor forward and reflected power during the RF heating experiments

A thermal MR applicator was constructed to be operated with a 300 MHz (*B*_*0*_ = 7.0 T) MR scanner (Siemens Healthcare, Erlangen, Germany). The applicator consists of eight antenna building blocks placed in a symmetrical arrangement around a cylindrical phantom (Fig. 1a). The building blocks are driven by an 8-channel multi-transmit system of the MR scanner (TX-Array, Siemens Healthcare, Erlangen, Germany) utilizing an 8 x 1 kW array of pulsed RF power amplifiers (Stolberg HF Technik AG, Stolberg-Vicht, Germany) (Fig. 1b) [20]. The pulsed power amplifier allows to be driven at P_max_ with 10 % duty cycle and a pulse length of 5 ms resulting in an average power of 800 W. For a short period the duty cycle can be increased to >20 % doubling the available average power. Each antenna building block consists of a *λ*/2 electric dipole antenna in a bow tie shape and a high permittivity dielectric (Deuteriumoxide (D_2_O), isotopic purity 99.9 atom % D, *ε*_*r*_ = 80) [20]. The use of D_2_O as a substrate facilitates ^1^H MRI free of signal contributions from deuterium since the gyromagnetic ratio of ^2^H deviates from hydrogen. This prevents artefacts due to limitations in the dynamic imaging range caused by strong *B*_*1*_^*+*^ field contributions in the vicinity of the electric dipole antenna. The high permittivity of the substrate allows shortening of the electric dipole length of the antenna since the wavelength in this medium is reduced by approximately √*ε*_*r*_. This enables antenna positioning around the human head with an application in the human brain. The Poynting vector of such arrangement is directed towards the target region for RF heating and MRI, with the *E*-fields being parallel to the electric dipole which are aligned with the direction of the static magnetic field *B*_*0*_ (z-direction). In the radio frequency range induced temperature elevations are predominantly caused by *E*-fields [23], which are the dominant factor of power absorption in tissue as expressed by the specific absorption rate (SAR):

1 $$ SAR=\frac{\sigma (f)\left|\overrightarrow{E}\right|{}^2}{\rho } $$

with *σ*(*f*) the frequency dependent electrical conductivity of tissue, *ρ* the tissue density and $\left|\overrightarrow{E}\right|{}^2$ the amplitude of the electric field vector. The *H*-fields perpendicular to *B*_*0*_ (x-y-direction) form the spin excitation field *B*_*1*_^*+*^ to be exploited for MRI and MRTh.

Even though certain ceramics (e.g., BaTiO_3_) can be used as a substrate with lower loss tangents than D_2_O, a liquid dielectric is preferred as a substrate in our implementation. An RF hyperthermia application with high RF power might lead to temperature elevations in the substrate caused by RF losses or thermal conductivity. The temperature dependence of the electromagnetic properties and hence the impedance of the substrate exhibits the risk to increase power reflections due to impedance mismatch during the cause of a treatment. A liquid dielectric offers the flexibility and benefit to be used in a temperature control circuit to stabilize the substrate temperature.

### Electromagnetic field simulations

**Fig. 2:**
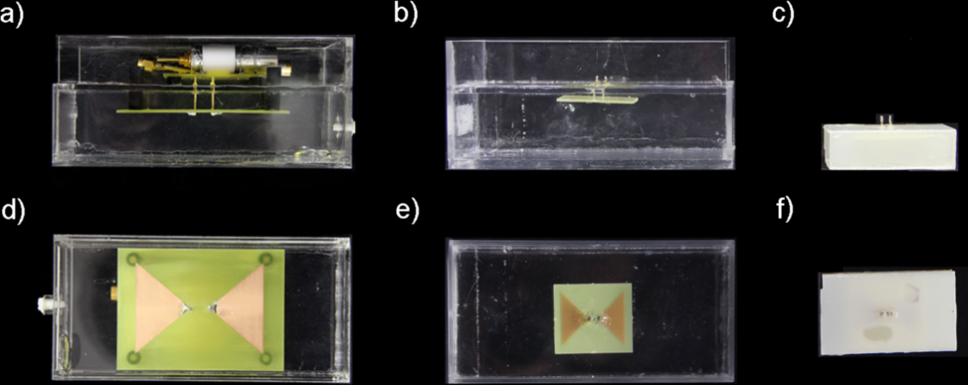
**a**-**f** Constructed bow tie building blocks used for RF heating in an integrated or hybrid system (**a**-**c**) sagittal and (**d**-**f**) coronal view. (**a**,**d**) 300 MHz setup with the dimensions (150 × 70 × 40) mm^3^ and D_2_0 as a substrate. (**b**,**e**) 500 MHz setup with the dimensions (150 × 70 × 40) mm^3^ and (c,f) with the dimensions (70 × 40 × 20) mm^3^, while deionized water has been used as a substrate

To investigate the EMF and SAR distribution, finite integration technique simulations were performed with CST Microwave Studio 2012 (CST, Darmstadt, Germany). Employing higher RF frequencies (*f* > 300 MHz) allows to reduce the size of the power absorption hotspot along x-, y- and z-dimension. In the x-y-plane (Fig. 1b) the hotspot size is determined by the RF wavelength in a lossy dielectric or brain tissue. The hotspot dimensions along the z-direction can be reduced by decreasing the size of the dielectric at higher RF frequencies, which allows for smaller transmit elements along that axis or by the use of multiple antenna rings [24–26]. To quantify these effects, SAR distributions produced by two 8-channel setups consisting of RF antenna building blocks with a fixed substrate size of (150 × 70 × 40) mm^3^ were examined at 300 MHz and 500 MHz (Fig. 2a, b, d, e). These configurations were benchmarked against a smaller substrate size of (70 × 40 × 20) mm^3^ at 500 MHz (Fig. 2c, f). For this purpose the antennas were placed around a cylindrical phantom (diameter = 180 mm, length = 250 mm) with tissue properties (*ε*_*r*_ = 75, *σ* = 0.72 S/m). The effective dimensions of deep lying hotspots were analysed using iso-contour calculations of the SAR distribution. For this purpose iso-SAR 25 %, iso-SAR 50 %, iso-SAR 75 % and an iso-SAR 90 % thresholds were derived for x-, y- and z-dimension based on the maximum point SAR value. SAR1g (1 g average, IEC 62407–1) was calculated for human voxel “Ella” from the virtual family [27].

### Hybrid RF heating at 500 MHz

To validate the results derived from the numerical simulations and to demonstrate that the RF frequency used for targeted RF heating may differ from the magnetic resonance frequency used for MRTh, RF heating experiments were performed at 500 MHz while MRI and MRTh was conducted at 7.0 T (300 MHz). The RF heating setup of the hybrid approach is shown in Fig. 1b and consists of a signal generator (Rhode & Schwarz, Munich, Germany), a class A linear RF power amplifier (RFPA) module (LZY-1, Mini-Circuits, New York, USA) capable to provide a continuous wave output power of ~ P_out_ = 140 W at f = 100–500 MHz and two power reflection meters (Rhode & Schwarz, Munich, Germany). One power meter was used to monitor transmitted and reflected power during the experiments, while the other power meter was used together with a 30 dB damping and a 50Ω termination to calibrate the RF losses introduced by the RF cable connecting the RFPA and the antenna. A single bow tie dipole antenna (150 × 70 × 40) mm^3^ was applied to induce RF heating in a custom made cylindrical phantom (diameter = 180 mm, length = 250 mm) (Fig. 2b, e). The phantom has an outer shell of polymethylmethacrylate filled with a mixture of deionized water, copper sulfate (Cu(II)-SO_4,_ 0.74 g/l) and sodium chloride (NaCl, 3.33 g/l) to resemble conductivity and permittivity of brain tissue. Agarose (C_12_H_18_O_9_, 20 g/l) was used as a gelling agent to suppress thermal convection. The resulting electromagnetic properties (*ε*_*r*_ = 72, *σ* = 0.75 S/m) were measured based on impedance measurements with a network analyzer (Agilent 4296B, Santa Clara, CA, USA). For RF heating an input power of 70 W was calibrated at the antennas feeding point. An RF heating duration of 12 min was used. To support ^1^H MRTh, the setup was placed inside a birdcage volume RF coil (Siemens Healthcare, Erlangen, Germany, *f* = 300 MHz, inner diameter = 34 cm). As a high permittivity substrate for the RF heating antennas at 500 MHz deionized water was used instead of D_2_0. In this particular arrangement the *B*_*1*_^*+*^ amplitude in the substrate and the phantom exhibits signal variations that fall well within the dynamic imaging range while the electromagnetic properties (RF losses and permittivity) of both D_2_0 and H_2_0 are comparable. MRTh was conducted using the proton resonance frequency shift (PRFS) method with a dual gradient echo technique (TE_1_ = 22.6 ms, TE_2_ = 6.34 ms) and a spatial resolution of (1.9 × 1.9 × 5) mm^3^ [28, 29]. *B*_*0*_ drift (0.02 ppm/h) was compensated with a vegetable oil sample as a reference [30].

### Thermal simulations

For a more realistic scenario, EMF simulations were performed with the voxel model “Ella” from the virtual family [27] at 300 MHz, 500 MHz and 1 GHz. All electromagnetic properties of the voxel model are based on [31]. The values for brain grey and white matter and blood are surveyed in Table 1. To demonstrate an advantage of even higher RF frequencies for a more localized RF heating, a 20-channel antenna array consisting of electrical dipole antennas (length = 142 mm) driven at 1 GHz was implemented and compared with the 300 MHz and 500 MHz setups. SAR distributions were calculated for an in-phase (0° phase shift between channels) phase setting. The power loss distribution was used as an input for the thermal simulations. Thermal simulations were conducted using CST MPhysics (CST, Darmstadt, Germany) solving the bioheat transfer equation:

**Table 1:** Electromagnetic and thermal properties of brain tissue

Tissue	Permittivity			Electrical conductivity [S/m]			Density [kg/m^3^]	Heat capacity [J/kg/°C]	Thermal conductivity [W/m/°C]	Heat transfer rate [ml/min/kg]	Heat generation rate [W/kg]
	300 MHz	500 MHz	1 GHz	300 MHz	500 MHz	1 GHz					
White matter	43.8	41	38.6	0.41	0.47	0.62	1041	3583	0.48	212	4.32
Grey matter	60	55.8	52.3	0.69	0.78	0.99	1045	3696	0.55	764	15.54
Blood	65.7	63.3	61.1	1.32	1.38	1.58	1060	3900	0.52	10000	0

Electromagnetic and thermal properties for 300 MHz, 500 MHz and 1 GHz of brain grey matter, brain white matter and blood used in the electromagnetic simulations with the human voxel model “Ella” [28]. All other material parameters used in the electromagnetic field simulations (e.g., skin or fat) can be found in [29]

2 $$ {c}_t{\rho}_t\frac{\partial T}{\partial t}=\nabla k\nabla T+{\rho}_t(SAR)+A-{\rho}_b{c}_b{\rho}_t{F}_t\left(T-{T}_b\right) $$

with the specific heat of tissue *c*_*t*_ in [*J*/(*kg* ⋅ *°C*)], the tissue density *ρ*_*t*_ in [*kg*/*m*^3^], tissue temperature *T* in [*°C*], thermal conductivity of tissue *k* in [*W*/(*m* ⋅ *°C*)], the basal metabolic heat generation rate of tissue *A* in [*W*/*kg*], the blood density *ρ*_*b*_ = 1060*kg*/*m*^3^, the specific heat of blood *c*_*b*_ = 3900*J*/(*kg* ⋅ *°C*), the tissue heat transfer rate *F*_*t*_ in [*ml*/(min ⋅ *kg*)] and the blood temperature *T*_*b*_ = 37*°C*. The heat capacity and thermal conductivity of the phantom material was *c*_*phantom*_ = 3546*J*/(*kg* ⋅ *°C*) and *k*_*phantom*_ = 0.53*W*/(*m* ⋅ *°C*) respectively. Please note that the basal metabolic heat generation rate and the term related to blood perfusion in Eq.2 are not considered for the thermal simulations in the phantom. The head of the human voxel model “Ella” from the virtual family was used, with a voxel size of (2 × 2 × 2) mm^3^ and thermally adjusted parameters based on [31]. All thermal parameters can be found in [31], thermal values for grey and white matter of the brain and blood are summarized in Table 1. RF heating in the voxel model was simulated for a duration of 3 min with a constant input power of 400 W per applicator. This approach resulted in an effective input power of 50 W per antenna at 300 MHz and 500 MHz and 20 W per antenna at 1 GHz. RF antenna and RF component losses were not taken into account. A water bolus of a fixed temperature of 20 °C was used to cool the surface. The effective dimensions of deep lying temperature hotspots were analysed using iso-contour calculations of the temperature difference with regards to baseline temperature (37 °C). For this purpose iso-Temperature 90 %, iso-Temperature 75 %, iso-Temperature 50 % and iso-Temperature 25 % thresholds were derived based on the maximum overall temperature increase.

## Results and discussion

### Thermal MR applicator and antenna design

**Fig. 3:**
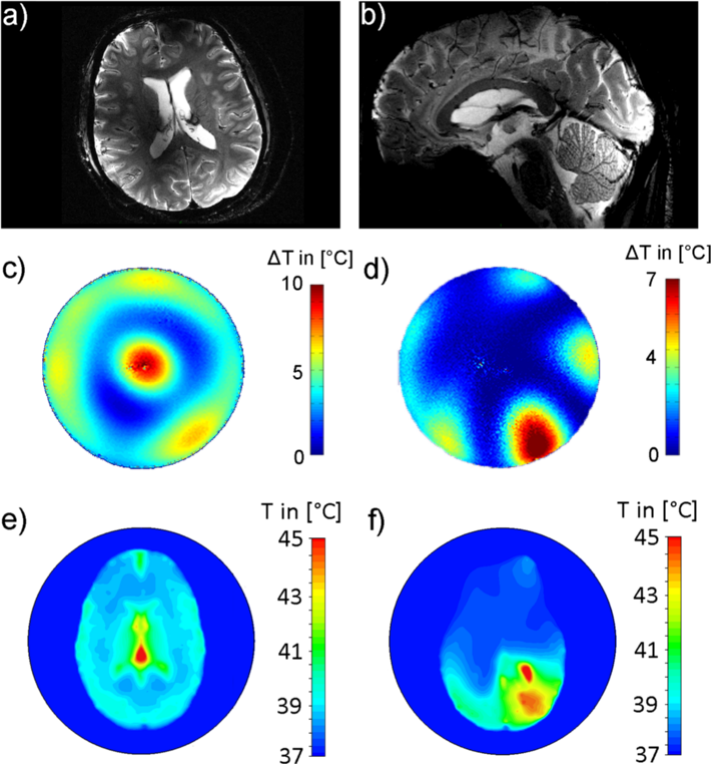
**a**-**b** MR images of the human brain acquired with the thermal MR 8-channel applicator at 7.0 T. A gradient echo technique is shown with a spatial resolution of (0.5 × 0.5 × 2.0) mm^3^. **c** MRTh maps of an RF heating experiment on a cylindrical phantom using the applicator to focus SAR in the center of the phantom and to acquire MR images to be used for the MRTh method. The pulsed power RF amplifier of the MR system was used at 300 MHz (^1^H proton excitation frequency at 7.0 T) to induce a temperature difference of ~11 °C in the center of the phantom after 3 min of RF heating. **d** Demonstration of 2D hotspot steering feasibility of the given setup with RF phase modulation between the channels [20]. A temperature of >7 °C in the vicinity of the phantom could be reached for an RF heating duration of 2 min. **e**-**f** Thermal simulations in human voxel model “Ella” [27] in order to demonstrate the capabilities of the applicator to generate a hotspot in (**e**) the center of the brain and (**f**) the periphery applying the same phase setting as in (**d**)

**Fig. 4:**
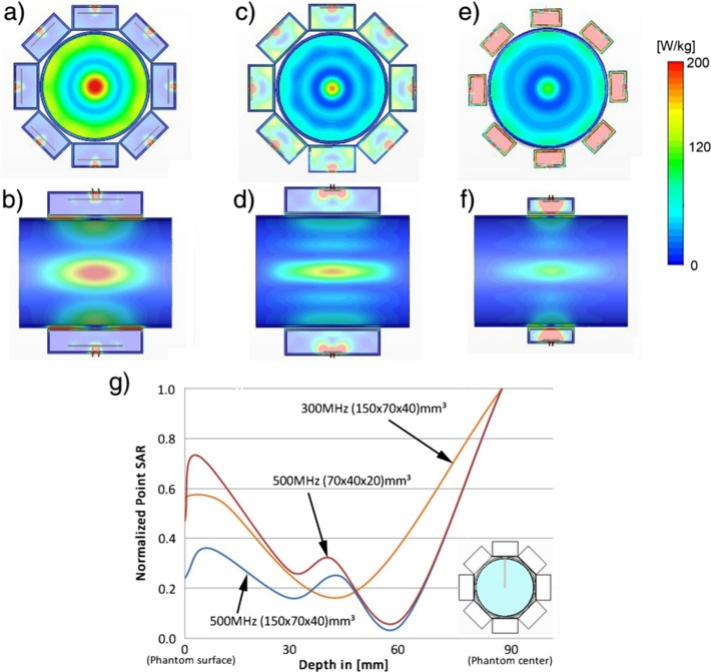
Comparison of the simulated SAR distributions for three 8-channel bow tie dipole antenna configurations and a total input power of 400 W (50 W per antenna). (**a**-**b**) Bow tie building block at 300 MHz with dimensions (150 × 70 × 40) mm^3^, (**c**-**d**) bow tie building block at 500 MHz with dimensions (150 × 70 × 40) mm^3^ and (**e**-**f**) a smaller sized (70 × 40 × 20) mm^3^ bow tie building block at 500 MHz. The local SAR hotspot size in the center of the phantom was significantly reduced at 500 MHz. A reduction in the length of the dielectric at 500 MHz (**e**-**f**) yielded smaller hotspot dimensions along the main axis of the bow tie antenna. (**g**) Comparison of the normalized point SAR derived from electromagnetic field simulations along a line from the surface to the center of a cylindrical phantom for three 8-channel arrangements of the building blocks. For all configurations the antenna building blocks are driven in phase (Ch1-8 = 0°) to generate a hotspot in the center of the phantom

The thermal MR 8-channel applicator supports high spatial resolution MRI at 7.0 T (Fig. 3a-b), MRTh (Fig. 3c-d) and targeted RF heating (Fig. 3c-d) in a cylindrical phantom mimicking brain tissue properties and the size of a human head [20]. The RF power of the MR system is sufficient to increase the temperature by approximately 11 °C in 3 min (Fig. 3c) [20]. The multi-channel transmit system of the MR scanner can be used to deliberately alter and steer the location of the hotspot (Fig. 3d) [20]. Thermal simulations in a human voxel model demonstrated that these phase settings can be reproduced to generate a hotspot in the center (Fig. 3e) and in the vicinity (Fig. 3f) of the human brain. The use of higher RF frequencies for targeted RF heating was found to decrease the hotspot size (Fig. 4). At 500 MHz (Fig. 4c-f), the dimensions of the SAR hotspot are significantly reduced (Table 2) from an iso-SAR 90 % value of (18 × 18 × 41) mm^3^ at 300 MHz to an iso-SAR 90 % value of (10 × 10 × 40) mm^3^ for the same antenna building block size (Fig. 4a-d, Table 2). Iso-SAR 75 % was (29 × 29 × 70) mm^3^ for 300 MHz and (17 × 17 × 73) mm^3^ for the same antenna building block size at 500 MHz (Table 2). Since the RF wavelength is shortened at 500 MHz, the dipole antenna and size of the dielectric can be reduced to enable smaller building block elements with a size of (70 × 40 × 20) mm^3^ (Fig. 4e-f) as compared to the larger elements (size = (150 × 70 × 40) mm^3^) used at 300 MHz and 500 MHz (Fig. 4a-d). The volume reduction of the building block by a factor of >7, in particular along the main axis of the bow tie antenna aligned with the z-direction, resulted in a smaller hotspot size (−32 %) along that axis (Table 2). An iso-SAR 90 % of (10 × 10 × 28) mm^3^ and an iso-SAR 75 % of (17 × 17 × 48) mm^3^ were found for the 8-channel setup (Fig. 4e-f, Table 2). The constructed bow tie building blocks are shown in Fig. 2 including a 300 MHz antenna (Fig. 2a, d), the 500 MHz counterpart (Fig. 2b, e) and the smaller 500 MHz version (Fig. 2c, f). The smaller RF antenna building block has the handicap of increased surface SAR values and a reduced absolute SAR value of 116 W/kg at the center of the phantom as compared to 176 W/kg at 500 MHz and 216 W/kg at 300 MHz for an input power of *P*_*in*_ = 400 W (Table 2). However its smaller size allows for more elements to be placed around the object which holds the promise to potentially reduce surface SAR hotspots, to increase the degree of freedom for transmission field shimming and hotspot steering and to boost the number of receive elements for higher signal-to-noise ratio (SNR) and accelerated acquisitions in an MRI application [32].

**Table 2:** RF heating performance of 8-channel applicator designs at 300 MHz and 500 MHz

Frequency [MHz]	Dimensions [mm^3^]	Central SAR [W/kg]	iso-SAR 90 % [mm^3^]	iso-SAR 75 % [mm^3^]	iso-SAR 50 % [mm^3^]	iso-SAR 25 % [mm^3^]
300	150 × 70 × 40	216	18 × 18 × 41	29 × 29 × 70	45 × 45 × 111	65 × 65 × 157
500	150 × 70 × 40	176	10 × 10 × 40	17 × 17 × 73	27 × 27 × 138	35 × 35 × 182
500	70 × 40 × 20	116	10 × 10 × 28	17 × 17 × 48	26 × 26 × 83	25 × 25 × 155

Maximum absolute central point SAR for a total input power of 400 W and hotspot size described by iso-SAR 90 %, iso-SAR 75 %, iso-SAR 50 % and iso-SAR 25 % along the x, y and z axis for transmit frequencies of 300 MHz and 500 MHz and for antenna building block with different dimensions

### Hybrid RF heating at 500 MHz

**Fig. 5:**
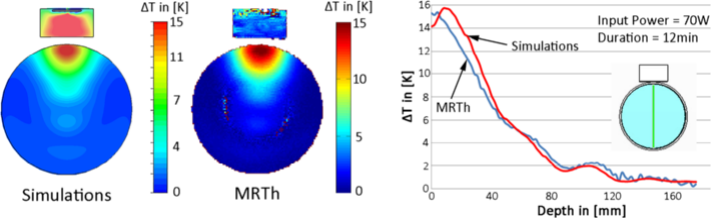
Comparison of the simulated temperature distribution (*left*) of a single bow tie antenna driven at 500 MHz in an axial slice of a cylindrical phantom versus MRTh measurements (*middle*). For simulations and experiments an RF heating paradigm of 70 W input power and a duration of 12 minutes was employed. Relative temperature distribution (*ΔT*) (*right*) along a central line (marked in green in the inlay showing the setup). A good agreement was found between temperature simulations (*red line*) and MRTh (*blue line*)

The results of the RF heating experiments at 500 MHz and MRI at 300 MHz are summarized in Fig. 5 together with the thermal simulations derived from a virtual setup. The external RFPA is not MR compatible and was positioned in the operator room, which made the use of long RF cables necessary to connect the RFPA with the RF antenna. This connection introduced cables losses of −2.7 dB at 500 MHz, resulting in a required power output of 130 W at the RFPA to reach an input power of 70 W at the antenna. Additional RF losses, which increase with RF frequency, resulted from the deionized water used as a dielectric with a measured electrical conductivity at 500 MHz of 0.14 S/m. However the RF power achieved was sufficient to generate a maximum temperature increase of ΔT > 15 °C after 12 min heating in simulations and measurements (Fig. 5). MRTh was performed without any imaging artefacts caused by the RF antenna building block. The measured temperature profile shows good correlation with the simulated data (Fig. 5). Benchmarking of thermal simulations against measurements yielded a temperature difference <2 °C for surface regions, which might be attributed to imperfect thermal modelling in these locations. In comparison, a temperature difference of less than 1 °C was found at depth (>40 mm) (Fig. 5).

### Thermal simulations in human voxel models

**Fig. 6:**
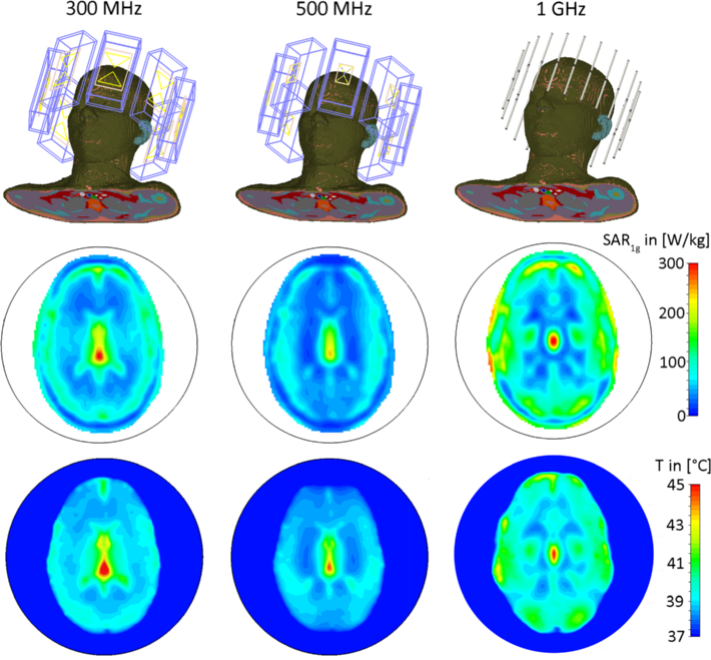
Comparison of the SAR and thermal distributions from electromagnetic and thermal simulations of three applicator configurations positioned around the head of human voxel model “Ella” [27] with frequency dependent electromagnetic and thermal properties adjusted based on [31]. The configurations include an 8-channel applicator at 300 MHz (*left*), an 8-channel applicator at 500 MHz (*middle*) and a 20-channel applicator at 1 GHz (*right*). All three applicators were driven with a total input power of *P*_*in*_ = 400 W, an in-phase (no phase shift between channels) phase setting, an RF heating duration of 3 min and a waterbolus temperature of T_B_ = 20 °C. Maximum SAR_1g_ in the center of the brain was 311 W/kg for 300 MHz, 240 W/kg for 500 MHz and 307 W/kg for 1 GHz. The maximum temperatures reached in the center of the brain were found to be *T*_*max*_ = 46.8 °C at 300 MHz, *T*_*max*_ = 44.5 °C at 500 MHz and *T*_*max*_ = 45.3 °C at 1GHz while surface regions exhibited temperatures below 43 °C (41 °C for 300 MHz, 39.3 °C for 500 MHz and 42.9 °C for 1 GHz)

The thermal simulation results obtained for an 8-channel applicator at 300 MHz, an 8-channel applicator at 500 MHz and a 20-channel applicator at 1 GHz positioned around the head of the human voxel model “Ella” are presented in Fig. 6. The hotspot was focused to the center of the brain to demonstrate, that targeted RF heating is not restricted to surface regions and can be performed for deep seated intracranial lesions. For an input power of 400 W and an RF heating duration of only 3 min, all setups were found capable to deliver sufficient energy absorption in the center of the brain (maxSAR_1g_ = 311 W/kg for 300 MHz, maxSAR_1g_ = 240 W/kg for 500 MHz and maxSAR_1g_ =307 W/kg for 1 GHz) in order to reach temperatures above 44 °C. The thermal simulations showed that after 3 min the 300 MHz setup reached a maximum temperature of *T*_*max*_ = 46.8 °C in the center of the human brain resulting in a temperature difference of *ΔT* = 9.8 °C versus baseline. This value correlates well with the temperature difference of *ΔT* = 10.7 °C measured previously in phantom experiments using the same RF heating paradigm [20]. At higher RF frequencies the maximum temperature reached was slightly lower with *T*_*max*_ = 44.5 °C for 500 MHz and *T*_*max*_ = 45.3 °C at 1 GHz. The difference versus the 300 MHz setup can be attributed to pronounced power losses in tissue at these frequencies leading to damping of the electromagnetic waves. While the maximum temperatures in the center of the brain are well above 44 °C for all configurations, surface regions showed temperature elevations below 43 °C (41 °C for 300 MHz, 39.3 °C for 500 MHz and 42.9 °C for 1 GHz) demonstrating a good selectivity of the approach. Time dependent changes in thermoregulation have not been modeled and might influence absolute achievable temperatures.

At higher RF frequencies smaller temperature hotspot dimensions were reached, indicating a more localized RF heating performance. At 300 MHz iso-temperature 90 % of (6 × 9) mm^2^, iso-temperature 75 % of (10 × 16) mm^2^, iso-temperature 50 % of (18 × 50) mm^2^ and iso-temperature 25 % of (56 × 79) mm^2^ hotspot size was observed for the axial plane (Fig. 6). For 500 MHz the hotspot dimensions were reduced to iso-temperature 90 % of (5 × 10) mm^2^, iso-temperature 75 % of (9 × 22) mm^2^, iso-temperature 50 % of (14 × 44) mm^2^ and iso-temperature 25 % of (29 × 64) mm^2^ while at 1 GHz the hotspot size was only iso-temperature 90 % of (2 × 8) mm^2^, iso-temperature 75 % of (5 × 14) mm^2^, iso-temperature 50 % of (11 × 22)mm^2^ and iso-temperature 25 % of (20 × 34) mm^2^ (Fig. 6).

All three antenna configurations can potentially be used for thermal MR using 7.0 T, 11.7 T or 23.5 T proton MR frequency for targeted RF heating, MRI and MRTh. The necessary average power of 400 W as being used in the simulation setup can be reached using the standard RFPA of the MR system as previously demonstrated in 300 MHz RF heating experiments [20]. A hybrid approach that makes good use of external RFPAs driven at a higher RF frequency (*f* ≥ 300 MHz) for RF heating together with a MR system operating at *B*_*0*_ = 1.5 T (64 MHz) or *B*_*0*_ = 3.0 T (128 MHz) for MRI and MRTh is also feasible.

## Conclusion

This study outlines the physical and technical underpinnings of a non-invasive RF hyperthermia device utilizing the MR spin excitation frequency for controlled RF heating (thermal MR). EMF simulations and experiments demonstrate the feasibility of an 8-channel applicator for MR imaging, MR thermometry and controlled targeted RF heating at 7.0 T. Our experiments demonstrated that the pulsed multi-channel transmit system of a 7.0 T MR scanner supports targeted RF heating and provides enough power (P_avg_ > 400 W) to induce a temperature increase of ~10 °C in 3 min in the center of a head sized phantom. Our EMF simulations and experimental verifications [20, 29] demonstrate that this system is able to provide >15 % higher power absorption values in the target region for the same input power as compared to current non MR guided clinical RF hyperthermia systems in the head and neck region, which are able to rise tumor temperatures up to 43 °C with average input power levels of ~300 W [33, 34]. Modification of the RF phases applied allowed for hotspot steering to a deliberately selected location.

Our physics considerations, EMF simulations and preliminary experimental results show that higher RF frequencies allow for a more localized targeted RF heating approach, confirming previous studies [17, 19, 20]. Here the temperature hotspot dimensions could be further reduced at 500 MHz and 1 GHz versus the 300 MHz configuration. Three applicator designs were presented, which can potentially be utilized for thermal MR at 300 MHz, 500 MHz and 1 GHz. All configurations are MR compatible and can be also integrated in a hybrid approach equipped with an external RFPA used for RF heating while MRI is being conducted at lower fields (*B*_*0*_ = 1.5 T, 3.0 T). In particular, our studies demonstrated the feasibility of using an external RFPA for transmission at 500 MHz in conjunction with MRI and MRTh performed at 300 MHz (*B*_*0*_ = 7.0 T).

In comparison to a hybrid approach, thermal MR has the benefit of making additional RF hardware (RFPA, RF electronics, filters, antennas) or software to drive these components obsolete while adding another therapeutic dimension to a diagnostic MRI device. The use of many element local transmit/receive antennas at ultrahigh fields offers potential signal-to-noise ratio enhancements [35–38]. These enhancements can be translated into spatiotemporal resolution improvements [39, 40] which are beneficial for MR temperature mapping during RF heating interventions. While being important to dynamically control the desired temperature distribution by adjusting the RF pulses played out at the antennas, reliable temperature information is crucial for a thermal dose characterization in locations of intracranial lesion as well as in remote healthy tissue. Although E-field or SAR distributions cannot be measured directly with MR, local SAR can be estimated using MR prior to an RF heating procedure [41]. This allows for a control and adjustment of the treatment planning parameters of the applicator prior to the treatment. During an RF hyperthermia treatment, MRTh can function as a tool to retrospectively estimate SAR and correct the temperature distribution via amplitude and phase modulation of the RF pulses [42, 43]. Here the selectivity of the method to provide an accurate thermal dose to a target region is yet to be determined *in-vivo*. Dynamically played out RF pulses can be optimized based upon the geometry and shape of intracranial lesions using adapted algorithms proposed to manage local SAR hotspots in parallel transmit MR applications [44–46]. The lessons learned from RF induced heating of conductive implants at 7.0 T [29, 47] can be also put to good use to advance targeted RF heating at 300 MHz.

Our findings suggest that at 1 GHz sufficient energy can be deposited in the center of the human brain. This result can be exploited for targeted RF heating, but also renders human *in-vivo* MRI at 23.5 T or human *in-vivo* electron paramagnetic resonance (EPR) at 1 GHz and above feasible from an electrodynamic standpoint. While a single transmission element faces an increased power absorption in surface regions at higher RF frequencies, the reduction of the transmitted wavelength enables a higher density placement of the antennas without deteriorating element decoupling. Exploiting this physical advantage, our simulations showed that the number of dipole antennas can be extended to 20 with decoupling values < −13 dB. At the same time a substrate, which might introduce losses becomes redundant at such high frequencies. The presented 20-channel electric dipole array at 1 GHz (23.5 T) generates absolute *B*_*1*_^*+*^ values of ~11μT/√kW in the center of the human brain while local SAR (1 g average) values are reduced at 1 GHz by a factor of ~2 as compared to 300 Mhz since the RF power is spread more evenly around the surface of the head [48]. These results provide encouragement for whole-body MR systems with *B*_*0*_ ≥ 7.0 T which is in alignment with potential future developments of UHF MRI. A recent report of the National Research Council on high magnetic field science and its application forwarded a call for a 20.0 T wide bore MR system [49]. This development is inspired by the progress at 7.0 T,by the early experience with small animal MR at 21.1 T and the advances in ultrahigh field magnet technology [50, 51]. The requirements of thermal MR are likely to pave the way for further advances in MR technology and MR systems design. With appropriate multi transmit systems that offer more than 8 transmission channels, an optimistically-inclined scientist might envisage the implementation of high density transceiver arrays to break ground for a many element, two-dimensional applicator allowing improvements in imaging performance [36] and hotspot focusing along z-dimension [24–26].

To summarize, the strong rationale for the use of non-invasive hyperthermia as part of a multimodal treatment for patients with glioblastoma asks for innovations [6, 9]. Non-invasive RF hyperthermia approaches, that combine ultrahigh-field MRI together with controlled and localized RF heating, potentially offer another dimension to treatment efficiency and control [20]. Such system, employed for local RF hyperthermia as an alternative additive treatment for glioblastoma multiforme might be able to improve local control [5] by “fighting fire with fire” [9]. Interventions are not limited to an adjuvant to radiotherapy alone, temperature driven targeted drug and contrast agent delivery in conjunction with diagnostic MR imaging and spectroscopy might improve chemotherapeutic approaches with increased drug concentrations at tumor site and reduced drug side effects [52–56]. Applications may not be limited to the brain but could be extended to other body regions and disease models and might help to understand temperature dependent bio- and physiological effects *in-vivo*. A limitation of thermal MR at UHF (B0 ≥ 7.0 T) are the current costs of the MR system, which might limit RF hyperthermia of the brain to established high-volume treatment centers.

To conclude, the opportunities and capabilities of thermal magnetic resonance for RF hyperthermia interventions are intriguing and in a creative state of flux. Bringing these interventions and therapies into the clinic remains a challenge. A story worth following since the implications feed into a broad spectrum of MR physics, biomedical engineering, oncology, surgery, radiology and other related fields of basic research and clinical science.

## Abbreviations

Λ: Radio frequency wavelength
B_0_: Static magnetic field used for magnetic resonance imaging
B_1_^+^: Magnetic resonance spin excitation field
CT: Computed tomography
EMF: Electromagnetic fields
MRI: Magnetic resonance imaging
MRTh: MR thermometry
PRFS: Proton resonance frequency shift
RF: Radio frequency
RFPA: RF power amplifier
SAR: Specific absorption rate
SNR: Signal-to-noise ratio
